# Oxygen-dependence of mitochondrial ROS production as detected by Amplex Red assay

**DOI:** 10.1016/j.redox.2018.04.014

**Published:** 2018-04-14

**Authors:** Vera G. Grivennikova, Alexandra V. Kareyeva, Andrei D. Vinogradov

**Affiliations:** Department of Biochemistry, School of Biology, Moscow State University, Moscow 119234, Russian Federation

**Keywords:** AR, Amplex Red, 10-acetyl-3,7-dihydrophenoxazine, FCCP, carbonyl cyanide 4-(trifluoromethoxy)phenylhydrazone, FMN and FMNH_2_, flavin mononucleotide, oxidized and reduced forms, respectively, HP, horseradish peroxidase, Res and ResH_2_, resorufin and dihydroresorufin, respectively, RET, respiratory complex I-mediated reverse electron transfer, ROS, reactive oxygen species, SMP, submitochondrial particles, SOD, superoxide dismutase, Hydrogen peroxide, Amplex Red, Resorufin, Respiratory chain, Respiratory complex I, Mitochondria

## Abstract

The initial rates of superoxide *plus* hydrogen peroxide (ROS) generation by intact or permeabilized rat heart mitochondria and coupled inside-out bovine heart submitochondrial particles (SMP) oxidizing NAD-dependent substrates, NADH, and succinate were measured by detecting resorufin formation in the Amplex Red assay at various oxygen concentrations. Linear dependences of the initial rates on oxygen concentration within the range of ~125–750 μM were found for all significant mitochondrial generators, i.e. the respiratory complexes and ammonium-stimulated dihydrolipoamide dehydrogenase. At lower oxygen concentrations upon its decrease from air saturation level to zero, the time-course of resorufin formation by SMP catalyzing coupled oxidation of succinate (the total ROS production by respiratory complexes II and III and by the reverse electron transfer (RET)-mediated by complex I) also corresponds to the linear dependence on oxygen with the same first-order rate constant determined in the initial rate studies. Prolonged incubation of SMP generating succinate-supported complex I-mediated ROS affected neither their NADH oxidase nor ROS generating activity. In contrast to SMP significant deviation from the first-order oxygen dependence in the time-course kinetics during coupled oxidation of succinate by intact mitochondria was evident. Complex I catalyzes the NADH:resorufin oxidoreductase reaction resulting in formation of colorless reduced resorufin. Hydrogen peroxide oxidizes reduced resorufin in the presence of peroxidase, thus showing its dihydroresorufin peroxidase activity. Combined NADH:resorufin reductase and dihydroresorufin peroxidase activities result in underestimation of the amount of hydrogen peroxide generated by mitochondria. We conclude that only initial rates of the mitochondrial ROS production, not the amount of resorufin accumulated, should be taken as the reliable measure of the mitochondrial ROS-generating activity, because of the cycling of the oxidized and reduced resorufin during Amplex Red assays fed by NADH and other possible reductant(s) present in mitochondria.

## Highlights

• The respiratory chain ROS production strictly linearly depends on oxygen concentration.
• Resorufin acts as electron acceptor for NADH-reduced complex I.
• Resorufin reductase distorts actual dependence of mitochondrial ROS production on oxygen.

## Graphical abstract

**Figure:**
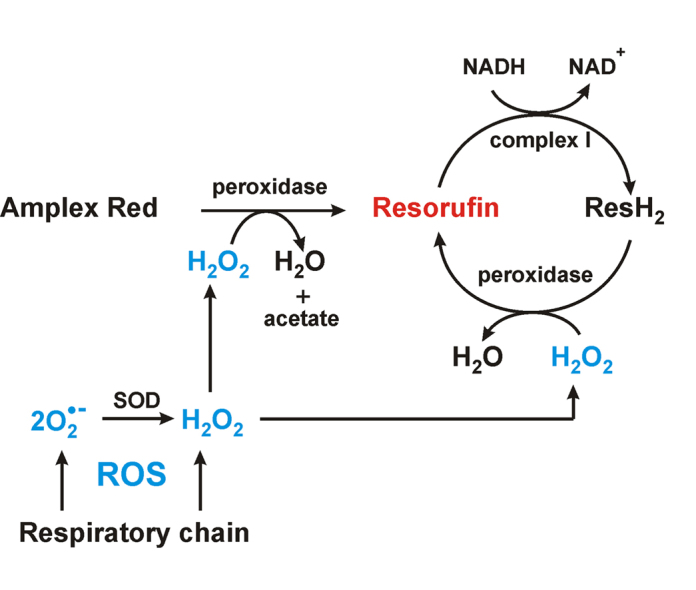

## 1 Introduction

Partially reduced oxygen (superoxide radical, hydrogen peroxide, hydroxyl radical) conventionally called reactive oxygen species (ROS) can result in deleterious oxidative stress if overproduced or serve as physiologically indispensable metabolites when present at their normal level. Since publication of seminal paper by Gershman et al. in 1954 [1] and discovery of hydrogen peroxide formation by antimycin-inhibited submitochondrial particles (SMP) [2], numerous reports have been published on general properties of mitochondrial ROS production, substrate donors, sites where they form, and their pathophysiological significance. Excellent accounts on those particular aspects reviewed by different research groups are available [3], [4], [5], [6], [7], [8], [9], [10]. Less attention has been paid to a dependence of ROS production on oxygen, an obligatory participant in the process, and somehow controversial data have been reported. Linear dependence of hydrogen peroxide production on oxygen by mitochondria isolated from various tissues, species, and isolated respiratory complex I on oxygen concentration have been reported [11], [12], [13], [14], [15], [16], whereas hyperbolic and substrate-donor- and respiratory state-dependent dependence have also been narrated for rat liver mitochondria [17], [18]. Previously, we found first order rate, i.e. linear dependence on oxygen, of succinate-supported energy-linked superoxide production by bovine heart SMP [19] as detected by reduction of acetylated cytochrome *c* [20] upon continuous oxygen consumption starting from normal atmospheric air saturation down to about 10 μM. Physiological concentrations of oxygen in various tissues in different metabolic states are diverse and significantly lower (5–10-fold) than that in air-saturated solutions [21], [22]. Therefore, the oxygen dependence of ROS production at its low concentrations seems to be particularly relevant for cell physiology.

Accurate determination of ROS in biological samples is far from trivial because of their inherent high and unspecific reactivity and very low steady-state concentrations [23]. Several methods have been developed for reliable measurements of ROS production [24], [25]. Amplex Red (AR) assay [26], [27] seems among most widely used for hydrogen peroxide detection. The reactions involved in H_2_O_2_ quantitative detection are following:

$$\begin{array}{c} \;\;\;\;\;{\mathrm{AR}+H}_{2}O_{2}\underset{\to }{\mathrm{peroxidase}}\;\mathrm{resorufin}\left(\mathrm{Res}\right)\;{+H}_{2}O+\text{acetate}\\ \text{Colorless},\;\;\;\;\;\;\;\;\;\;\;\;\;\;\;\;\;\;\;\;\;\;\;\;\;\;\;\;\;\;\;\;\text{Colored},\\ \mathrm{non}\;\mathrm{fluorescent}\;\;\;\;\;\;\;\;\;\;\;\;\;\;\;\;\;\;\;\;\;\mathrm{fluorescent} \end{array}$$

If catalytically competent peroxidase and superoxide dismutase (SOD) are present the stoichiometry (H_2_O_2_ + 2 superoxide)/Res is 1. Because of high absorption/fluorescence of Res and the relative specificity of the acetyldihydrophenoxazine peroxidase reaction, very low concentration of H_2_O_2_ can be detected, although several possible pitfalls due to involvement of required auxiliary enzymes (peroxidase and SOD) have been discussed [28], [29], [30], [31].

The original purpose of this study was to evaluate a dependence of heart mitochondria ROS production over a wide range of oxygen concentrations, particularly at its low concentrations. Our observations on AR assay prompted us to look closer at some properties of resorufin, which should be taken into account if used for mitochondrial ROS production.

## 2 Materials and methods

Rat heart mitochondria [32], [33] were prepared as described. Adult rats (3–6 months of age) were treated according to “The rules of research activity in biology, medicine, and other related areas” approved by the Russian Federation acts according to the international standards (GLP). Bovine heart inside-out coupled and activated SMP [34], [35] were prepared from bovine hearts obtained from slaughterhouse material in Moscow. Mitochondria and SMP were assayed in the standard reaction mixture composed of 0.25 M sucrose, 5 mM potassium phosphate, 10 mM KCl, and 0.1 mM EDTA (pH 7.5) at 30 °C. Other additions to the mixture are indicated in the figure legends. The data were analyzed with the assumption that no significant difference in the mitochondrial enzymes content, location, and their specific activities exist for *Bos taurus* and *Rattus rattus*. Where indicated, the mitochondria were permeabilized in the assay mixture by preincubation with alamethicin (40 μg/ml) and 2.5 mM MgCl_2_ for 1 min [36]. Hydrogen peroxide formation was measured photometrically with AR (10 μM) as formation of Res (ε_572–600_ = 54 mM^–1^ cm^–1^ [26]) in the standard reaction mixture supplemented with horseradish peroxidase (HP, 2 units/ml) and bovine erythrocyte SOD (6 units/ml). Total hydrogen peroxide-producing activity assayed with AR in the presence of SOD is the sum of the specific H_2_O_2_ generation *plus* half of the superoxide-producing activity. The hydrogen peroxide assays were calibrated by the addition of proper aliquots of H_2_O_2_ from freshly prepared stocks made by dilution of concentrated photometrically determined (ε_230_ = 81 M^–1^ cm^–1^) solution. Oxygen consumption was measured amperometrically with an oxygen-sensitive membrane-coated platinum electrode. AR, SOD, and HP were added to the standard oxygen assay samples at the same concentration as added to the ROS production assays; they did not affect oxygen consumption. The initial rates of succinate-supported reverse electron transfer activity (RET) catalyzed by SMP at different oxygen concentrations was measured aerobically in the standard reaction mixture supplemented with 5 mM succinate and 1 mM NAD^+^. ATP-dependent RET [34] was measured as before except for 3 mM ATP•Mg^2+^ and sodium sulfide (2 mM) (to prevent respiration) were added. Protein content was determined by the biuret procedure.

**Fig. 1:**
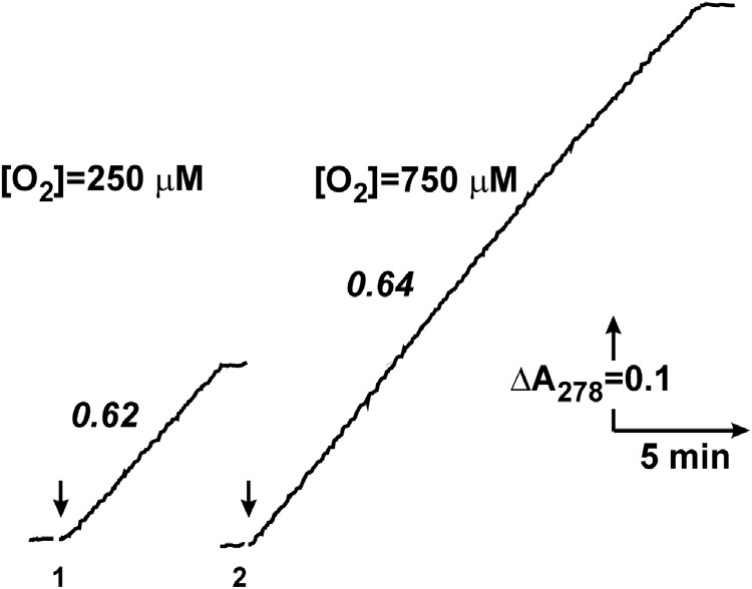
An example of determination of oxygen concentration in the standard reaction mixtures used throughout this study. Uncoupled (2 μM FCCP was added) oxidation of 5 mM succinate was traced as fumarate formation determined at 278 nm $\left(\varepsilon \frac{\mathrm{mM}}{278}=0.3\right)$. The reaction was initiated by the addition of SMP (0.15 mg of protein per ml) (indicated by arrows) to the closed spectrophotometric cuvette with air saturated (curve 1) and standard reaction mixture saturated by pure oxygen (curve 2). Figures on the traces in italic are the specific oxidase activities (μmol/min per mg of protein). Practically no fumarate formation was seen in a reaction mixture saturated by pure argon.

Reaction mixture with various concentrations of oxygen was made by mixing of proper volumes of the solutions saturated by either atmosphere air, or argon, or pure oxygen. The actual concentration of oxygen in thus prepared mixtures was determined as follows. Succinate (5 mM) oxidation by uncoupled (2 μM FCCP) SMP was traced by following zero-order fumarate formation at 278 nm $\left(\varepsilon \frac{\mathrm{mM}}{278}=0.3\right)$ up to abrupt termination of the reaction, and oxygen concentration was calculated as [O_2_] = 2 [fumarate]. An example of this procedure is illustrated in Fig. 1.

All data are presented as mean ± SEM of at least 3 independent experiments.

Succinate, malate, glutamate, Res, rotenone, FCCP, SOD from bovine erythrocytes (Cat. No. S7571) and from *Escherichia coli* (Cat. No S5639) were from Sigma-Aldrich (USA); NADH, HP (Cat. No. 195372) were from MP Biomedicals (USA); AR was from AnaSpec, Inc. (USA). NADH-OH was prepared essentially as described [37]. Other chemicals of highest purity available were from local suppliers.

## 3 Results

**Fig. 2:**
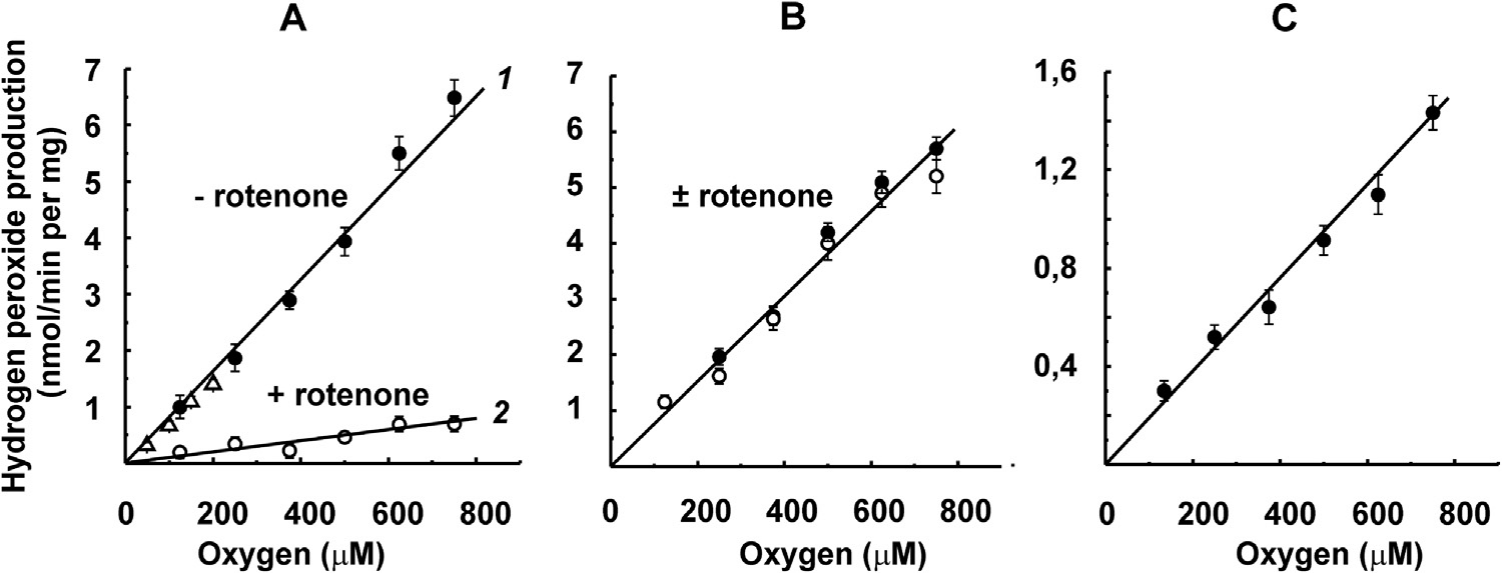
Initial rates of Res formation by coupled SMP (40 μg/ml) oxidizing (A), succinate (5 mM); (B), succinate (5 mM) and NADH (1 mM); and (C), succinate (50 μM) in the presence of 5 μM rotenone and 1.6 μM myxothiazol. Rotenone (5 μM) was added where indicated. Open triangles on line *1* in panel (A) depict the rates determined from the progress curve of Res formation as described in Fig. 5A.

Fig. 2 shows the initial rates of H_2_O_2_ *plus* superoxide generation at various oxygen concentrations by inside-out SMP, the preparation that produces ROS by the respiratory chain components only. Linearly dependent first-order reaction with oxygen was evident for ROS generation within the 125–750 μM range of O_2_. In accord with our previously published data [38], the highest rate was detected for complex I reduced by succinate via the RET as evident from strong decrease in the activity by rotenone, a specific inhibitor of the RET reaction (Fig. 2A). The addition of NADH did not affect the activity seen with succinate alone (Fig. 2B). It should be noted that we reported previously that NADH at high concentration decreases RET-induced superoxide generation [38]. The data in Fig. 2B where the rate of sum of hydrogen peroxide and superoxide production were measured under slightly different conditions revealed no inhibitory effect of added NADH. We left this apparent discrepancy for more detailed investigations in future experimentations. Complex II and the myxothiazol-insensitive site of complex III contributed less than 20% to the overall ROS production at all oxygen concentrations, although higher activity could be reached in agreement with previously reported data [35] at lower concentrations of succinate (Fig. 2C).

**Fig. 3:**
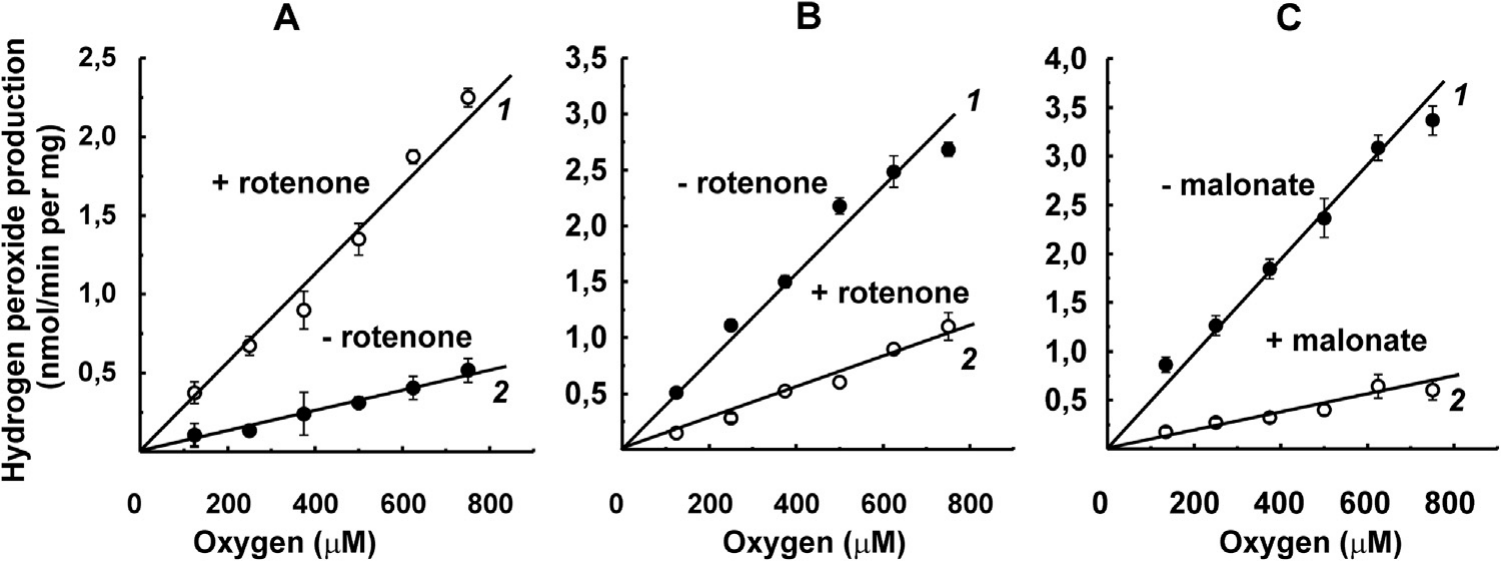
Initial rates of Res formation by intact rat heart mitochondria (50 μg/ml) oxidizing (A), glutamate and malate (5 mM each); (B), succinate (5 mM); (C), succinate (50 μM) in the presence of 5 μM rotenone and 1.6 μM myxothiazol. Rotenone (5 μM) and malonate (5 mM) were added where indicated.

**Fig. 4:**
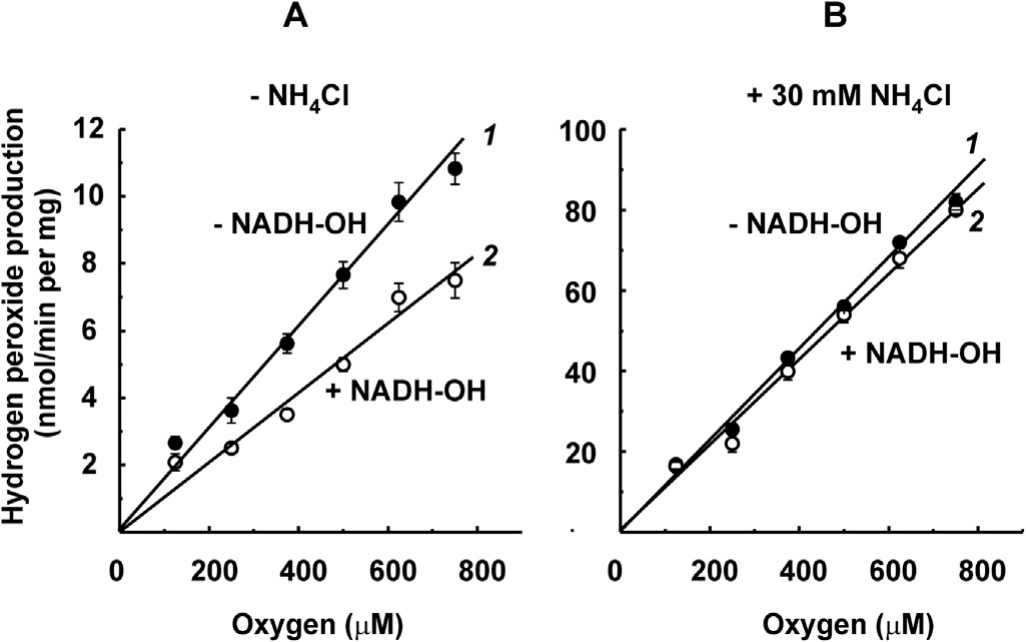
Initial rates of Res formation by permeabilized rat heart mitochondria. Mitochondria (30 μg/ml) were preincubated in the standard reaction mixture for 1 min in the presence of alamethicin (40 μg/ml) and 1 mM MgCl_2_. The reaction was initiated by the addition of 1 mM NADH. Where indicated mitochondria after permeabilization were preincubated with NADH-OH (70 nM) for 30 s. Ammonium chloride (30 mM) was added to the samples where indicated.

Linear dependence of the initial rates on oxygen concentrations within the 125–750 μM range was found under all the conditions employed for intact mitochondria, which contain other generators in addition to the respiratory chain components. ROS generation by intact mitochondria supplemented with glutamate and malate (NAD-dependent substrates) was strongly stimulated by rotenone, presumably because of complete reduction of complex I by endogenous NADH (Fig. 3A). The rate in the presence of rotenone became as high as that in the succinate-supplemented samples (Fig. 3B, curve 1), where the reaction was inhibited by rotenone because the RET pathway was abolished (Fig. 3B, curve 2). The respiratory chain component and matrix-located enzymes in intact mitochondria could not be directly reduced by added NADH because of the permeability problem. Reduction by NADH could be achieved using alamethicin-permeabilized mitochondria, which retain all matrix-located proteins [36]. The results obtained on permeabilized mitochondria are shown in Fig. 4. NADH (1 mM, the concentration approaching its physiological content in mitochondria) resulted in ROS generation at a rate higher than that detected in the succinate-reduced intact mitochondria, and the reaction was only partially inhibited by NADH-OH, a specific inhibitor of the NADH-binding site of complex I [37] (Fig. 4A). These results suggested that in addition to complex I, an NADH-reducible matrix component contributes to the overall ROS generation. It has been shown that α-oxoglutarate dehydrogenase produces hydrogen peroxide by its dihydrolipoyl dehydrogenase component [39], [40], [41], and the latter activity is strongly stimulated by ammonium [42]. Fig. 4B shows that in the presence of ammonium the overall NADH-dependent mitochondrial ROS generation was about 7-fold higher than that seen with NADH only; the partial contribution of complex I and other respiratory chain components became negligible and ammonium-activated ROS generation remained linearly dependent on oxygen. We noted that other than ROS-generating respiratory enzymes activity, overall succinate- and NADH-oxidase and aerobic succinate-supported or succinate-dependent ATP-supported RET were the same at 250 and 750 μM oxygen concentrations. Incubation of SMP with 200 μM H_2_O_2_ also did not affect those activities; accelerated decomposition of H_2_O_2_ in the presence of SMP was seen, most likely because of their small contamination by catalase.

**Fig. 5:**
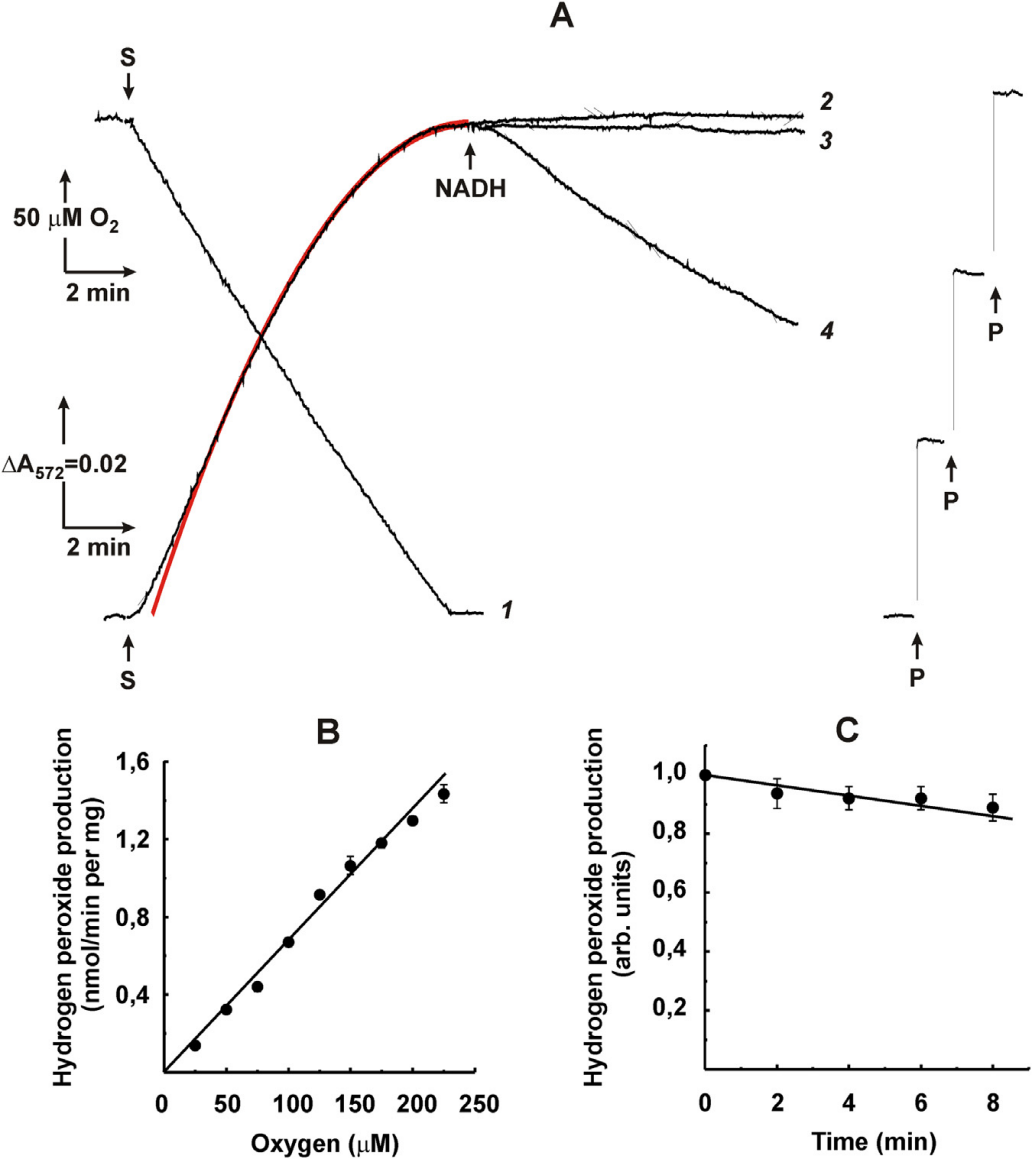
Time course of hydrogen peroxide production by coupled SMP (0.2 mg/ml) oxidizing succinate. (A), line 1, oxygen consumption initiated by 5 mM succinate (S) as measured amperometrically; line 2, Res formation; line 4, rotenone (5 μM) and NADH (100 μM) (indicated by arrow) were added after all oxygen was consumed (anaerobic conditions); line 3, NADH-OH (0.2 μM) was added before NADH; line 5 in red, calculated trace described by Eq. (4) (see text) with parameters: k_1_ = 0.0014 min^–1^ (first-order initial rate dependence depicted in Fig. 2A) and k_2_ = 30 μM/min (from zero-order rate constant of oxygen consumption, line 1). Calibration of the scale by additions of hydrogen peroxide (P, 0.5 μM each). (B), instant rates of the reaction obtained from derivatization of actual trace of Res formation shown in panel (A) plotted as a function of oxygen concentration. (C), initial rates of Res formation as a function of assay time. Aliquots (0.4 ml) were withdrawn from the sample assayed as depicted in panel (A), added to 1.6 ml of the standard reaction mixture supplemented by AR, HP, SOD, and 5 mM succinate, and the initial rates of hydrogen peroxide formation were measured. Arbitrary unit (1.0) corresponds to the specific rate of 1.4 nmol per min per mg.

To summarize the data reported above, it is safe to conclude that all heart mitochondria generators produce ROS simply proportional to oxygen concentration within the 125–750 μM range. It should be kept in mind, however, that the concentration range of oxygen employed so far is physiologically hyperoxic. As noted above, the real concentration of oxygen, being various in different tissues, is significantly lower than that in air-saturated solutions [21], [22]. We were particularly focused on examination the oxygen-dependence of ROS-generation at significantly lower, closer to physiological concentrations, i.e. in the system that gradually approaches anaerobiosis. To reach this goal, the time course of Res formation (equimolar to H_2_O_2_) and oxygen consumption upon oxidation of succinate by coupled SMP starting the reactions from air saturated reaction mixture was followed. As is well established, the generation of ROS by coupled respiratory chain components under these conditions mostly proceeds at complex I level [38], [43]. Fig. 5A shows actual tracings of zero-order oxygen consumption during coupled succinate oxidation (curve 1) and hydrogen peroxide production (curve 2) in a closed cuvette. The latter gradually ceased when the system approached anaerobiosis, and the amount of Res formed remained constant as long as the measurement was continued (curve 2). Stability of the RET-catalyzed ROS production by SMP was controlled in separate experiments where the succinate-supported reaction proceeded under the same conditions as in the progress curve registration. The initial reaction rates measured in aliquots withdrawn from the reaction mixture and assayed in air-saturated conditions remained constant for at least 8 min (Fig. 5C). The stability of the system and the linear calibration by externally added hydrogen peroxide shown on right side of Fig. 5A indicated that the only reason for curvature in Res formation resulted from gradual decrease in oxygen concentration. When the derivatives of the progress curves were plotted as a function of oxygen concentration, linear dependence was evident (Fig. 5B). Since the zero-order rate of oxygen consumption and the initial rate of hydrogen peroxide production (curve 2) were known, the time course for Res (H_2_O_2_) appearance could be described by the system of simple differential equations:

(1) *d* [H_2_O_2_]/*d* t = k_1_ • [O_2_]

(2) *d* [O_2_]/*d* t = 250 μM (initial concentration of oxygen) – k_2_ • t

where k_1_ stands for the first-order rate constant in H_2_O_2_ formation and k_2_ stands for the zero-order time dependence of oxygen consumption. Solution of this system of equations gives the theoretical course of H_2_O_2_ appearance as a function of time (t) for all positive values of [O_2_]:

(3) [H_2_O_2_]_t_ = 250• k_1_ • t – k_1_ • k_2_ • t^2^/2

and with absorption coefficient of Res (ε_572_ = 0.054 μM^–1^ cm^–1^) the time-dependent absorption change is:

(4) $${\Delta A}_{t}=0.054\;•\;\left(250•\;k_{1}{•t–k}_{1}{•k}_{2}{•t}^{2}/2\right)$$

**Fig. 6:**
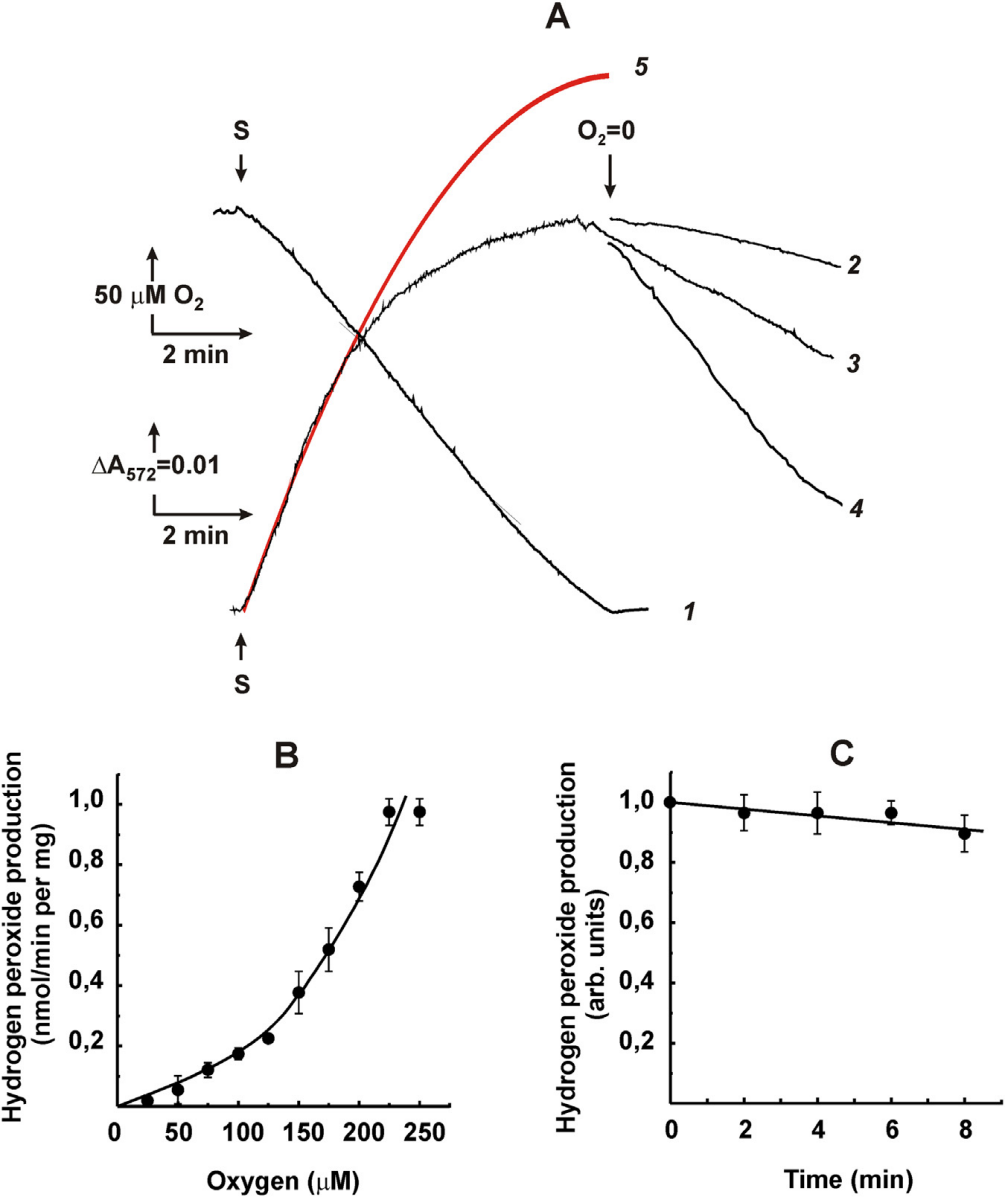
Time course of Res formation during oxidation of succinate by intact rat heart mitochondria (0.29 mg/ml). (A), actual traces of oxygen consumption (curve 1) and Res formation (curves 2–4). Kinetics of Res disappearance after the sample became anaerobic (indicated by arrow) are depicted by curves: 3, no further additions; 4, alamethicin (40 μg/ml) and MgCl_2_ (1 mM) were added; 2, as 4, 0.2 μM NADH-OH was added; line 5 in red, calculated trace described by Eq. (4) (see text) with the parameters: k_1_ = 0.0011 min^–1^ (first-order initial rate dependence depicted in Fig. 3B) and k_2_ = 33 μM/min (from zero-order rate constant of oxygen consumption, line 1). (B), instant rates of the reaction obtained from derivatization of actual trace of Res formation shown in panel (A) plotted as a function of oxygen concentration. (C), The initial rates of Res formation as a function of the assay time; see details in Fig. 5C. Arbitrary unit (1.0) corresponds to the specific rate of 1.0 nmol per min/mg.

The theoretical curve described by Eq. (4) shown in Fig. 5A (red line) satisfactorily fitted the experimentally observed tracing, and the experimental points shown in Fig. 5B corresponded to the linear dependence of ROS formation on oxygen over the full range of 0–750 μM O_2_ (Fig. 2A, open triangles). It should be noted that in the experiment depicted in Fig. 5, succinate was the only reductant of oxygen. When the experiment described in Fig. 5 was carried out with 1 mM NADH as oxidizing substrate, significant deviation from the curve theoretically constructed according to Eq. (4) (with proper rate constants) was observed. These results suggested that the system of Eqs. (1), (2) does not correctly describe the process of NADH-supported Res production. We proposed that NADH-reduced complex I might directly interact with Res. This proposal was indeed confirmed. When an anaerobic solution of NADH was added to the reaction mixture where succinate-supported H_2_O_2_ formation had been completed, a decolorization of Res was seen (Fig. 5A, curve 4), which was completely reversed up to the original level if oxygen was introduced simply by stirring of the open cuvette. The decolorization of Res by NADH was prevented by the specific inhibitor of complex I (NADH-OH) (Fig. 5A, curve 3). These observations suggested that Res acts as an autooxidizable reversible electron acceptor for NADH-reduced complex I. Next, the experimental setup for the progress curve analysis as described in Fig. 5A was applied to the reaction catalyzed by intact rat heart mitochondria (Fig. 6A). The initial rates of ROS production monitored in the preincubation experiments were as stable as in SMP, whereas the kinetics of Res formation progress curves was different. Spontaneous decolorization (reduction) of Res was seen once the system became anaerobic (curve 3). The rate of Res reduction was increased in a permeabilized sample (curve 4), and this reduction was partially prevented by inhibition of complex I (Fig. 6A, curve 2). When derivatives of the continuous registration curve shown in (A) were plotted as a function of oxygen concentration, strong deviation from linearity was obtained (B). When theoretical analysis of the progress curve described by Eq. (4) was applied, significant deviation from the experimentally observed kinetics was evident (curve 5 in red). The stability of the mitochondrial ROS generation demonstrated in Fig. 6C suggested that decrease in oxygen concentration was the only parameter affecting the time course of Res appearance. As a plausible explanation of the pattern shown in Fig. 6A and B, we proposed following. Significant redox cycling of Res starts when the system is coming to hypoxic conditions, which decreases the apparent amount of hydrogen peroxide detected at decreased oxygen concentration, and the contribution of the dye reduction became significant. Permeabilization of the inner mitochondrial membranes accelerated the reduction, most likely because increased permeability of Res to the matrix where its reduction by complex I occurs, as shown in Fig. 5A. Its incomplete inhibition by the specific inhibitor of complex I, NADH-OH (curve 2), shows that other reductant(s) of Res are located in the inner compartment of mitochondria.

**Fig. 7:**
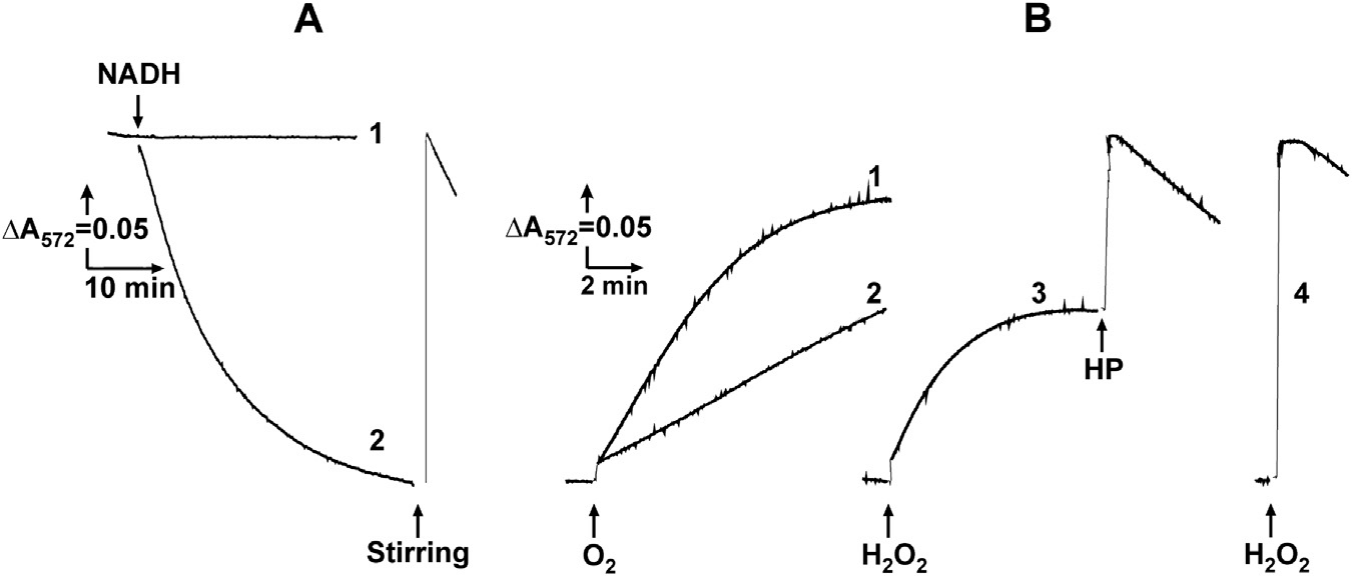
Reversible redox transformation of Res. (A) Res (4 μM) was added to the standard reaction mixture (2.4 ml) supplemented by SMP (0.4 mg/ml), succinate (5 mM), and rotenone (5 μM), and the mixture was incubated in a closed cuvette until all oxygen was consumed. NADH (100 μM) was added where indicated, and decolorization of Res was traced (curve 2). The cuvette was opened, stirred where indicated, closed, and further decolorization continued (curve 2). No change in absorbance was detected in samples where no NADH was added (curve 1). (B), Res was decolorized (reduced by NADH) as in panel (A), and a small amount of oxygen (10 μM, 0.1 ml of aerobic aqueous solution containing 25 mM potassium cyanide to prevent respiratory activity of SMP) and restoration of absorbance was followed (curve 1). *E. coli* SOD (cyanide-insensitive, 24 units/ml) was added (curve 2). ResH_2_ was recolorized by hydrogen peroxide (10 μM) (curves 3 and 4). HP (0.5 units/ml) was added where indicated (curve 3) or before hydrogen peroxide was added (curve 4).

To gain better insight into the redox reactivity of Res, further experiments were performed. The reduction of Res by complex I and other mitochondrial components are evident from the data shown in Fig. 5, Fig. 6. The question what is(are) its oxidation pathway(s) arose. Two-electron reduction of oxygen by reduced Res is expected to produce hydrogen peroxide. Because of spin restriction this reaction most likely proceeds via intermediate superoxide and formation of the reductant's radical. Whatever the specific mechanism is, the final product should be hydrogen peroxide. Interestingly, aeration of the mixture after complete reduction of Res as shown in Fig. 5, Fig. 6 did not produce hydrogen peroxide, which is expected as resulted from the additional oxidation of its acetylated derivative (AR) in the peroxidase reaction:

$$\begin{array}{c} \;\;\;\;\;\;\;\;\;\;\;\;\;\;\;\;\;\;\;\;\;\;{\mathrm{ResH}}_{2}{+O}_{2}\underset{\to }{\mathrm{spontaneously}}\;\;{\text{Res}+H}_{2}O_{2}\\ \;\;\;\;\;\;\;\;\;\;\;\;\;\;\;\;\;\;\;\;\;\;{\text{AR}+H}_{2}O_{2}\underset{\to }{\mathrm{peroxidase}}\;\;\;\;\;\;\;\;\;\;{\text{Res}+H}_{2}O\\ \text{Sum}:\;\;\;\;\;\;\;\;\;\;\;\;{\mathrm{ResH}}_{2}{+\text{AR}+O}_{2}\to 2\;\text{Res}+2\;H_{2}O \end{array}$$

when AR, SOD, and HP are present as they were in the samples shown in Fig. 5, Fig. 6. We proposed that the absence of additional hydrogen peroxide upon reoxidation of ResH_2_ can be explained if H_2_O_2_ is used in ResH_2_ oxidation by HP. This proposal was checked and confirmed in the experiments demonstrated in Fig. 7.

The reaction mixture containing SMP and Res was supplemented with succinate and incubated until all oxygen was consumed. Anaerobic NADH (100 μM) was then added and incubation was continued until all the Res was reduced. Extensive stirring of the mixture resulted in complete recovering of absorption (reoxidation of ResH_2_) (Fig. 7A). The kinetics of reoxidation was followed when it was induced by the addition of limited amounts of O_2_ (~10 μM) or H_2_O_2_ (10 μM). Either addition brought about time-resolved recolorization, which was slower and SOD-sensitive in the reaction with oxygen (Fig. 7B, curves 1 and 2, respectively) and faster with H_2_O_2_ (Fig. 7B, curves 3). The addition of HP to the sample with partially oxidized ResH_2_ resulted in its instant and complete oxidation, as it did if peroxidase was present in the sample before the addition of hydrogen peroxide (Fig. 7B, curves 3 and 4). NADH present in large excess (100 μM) continued the reduction of Res after its complete oxidation by HP. The results shown in Fig. 7 show unambiguously that ResH_2_ serves as a substrate for HP.

## 4 Discussion

Using the AR assay, we have confirmed and extended previously observed [11], [16], [19] simple linear dependence of ROS production by heart mitochondria and SMP oxidizing different substrates on oxygen concentration (Fig. 2, Fig. 3, Fig. 4). Either initial rate data (Fig. 2A, 125–750 μM range of oxygen concentration) or product progress curve analysis (Fig. 5B, 0–225 μM of oxygen concentration) concur with first-order oxygen reaction, thus suggesting that no specific binding sites or channels where oxygen binds or diffuse to meet its reductive counterpart exist in ROS generating components of the respiratory chain. A note should be made parenthetically that because of this linear dependence, the steady-state concentration of hydrogen peroxide inside and/or in the vicinity of mitochondria may serve as a simple physiologically relevant measure of intracellular oxygen concentration [44], [45].

We are unable to explain the data [17], [18] suggesting hyperbolic dependence of rat liver mitochondria ROS production on oxygen concentration and only short comments on the subject are worth making. No actual tracings of Res formation during “open flow respirometry and ROS measurements” were shown in these papers which makes difficult, if not impossible, to analyze the data, which clearly contradict the reports by several other independent groups [11], [12], [13], [14], [15], [16] including the findings reported here. The actual tracing of Res formation seems particularly needed for meaningful interpretation, because: (i) it is not clear how the rates of ROS production were calculated. The authors monitored fluorescence for 3 min after the liquid phase had reached a steady state, presumably several minutes after the reactions were initiated. It is not clear whether the fluorescence was then increased at constant rate; (ii) meaning of zero rate at zero oxygen concentration is not explained, whereas esterase activity of liver mitochondria producing Res independently of hydrogen peroxide have been demonstrated [46]. Half-maximal inhibition of the mitochondrial respiratory activity at transition to anaerobiosis (apparent K_m_ for oxygen) 2.7 and 3.6 µM O_2_ for State 3 and State 4, respectively, reported in Ref. [18] are also contradict to well established zero order oxygen consumption by respiring mitochondria down to about 0.3 µM oxygen.

Although cell biology literature is replete with circumstantial observation suggesting an increase of ROS production under hypoxia we found no any reports where this increase was directly measured as the reaction rate dependency on oxygen concentration. The cellular response to hypoxia under physiological conditions is extremely complicated process which evidently includes a number of regulatory known and unknown cascades. It should be emphasized that the data reported here concerns isolated heart mitochondrial ROS production exclusively and they can not be directly used for oversimplified interpretation of much more complex adaptive cellular and/or tissue behavior. Possible physiological significance of the mitochondrial ROS production have been discussed in our previous review paper [10] and this subject is beyond the scope of the present study.

We found no deteriorating effects of either high oxygen concentration or very high as compared to physiologically possible concentration of hydrogen peroxide (200 μM) on the respiratory chain activities when SMP were exposed at 30 °C for 30 min. This does not exclude some effects if the inner mitochondrial membrane is permanently exposed to ROS for a much longer time under physiological conditions.

It has been shown that FMN dissociates from its binding site of complex I when the enzyme is reduced [47], [48], [49]. Complex I is certainly reduced during the steady-state generation of ROS: its specific catalytic turnover in ubiquinone reduction or in reverse electron transfer is much higher than in the direct reaction with oxygen. The time-dependent decline in ROS generating activity seen in intact brain mitochondria upon coupled oxidation of succinate or NAD-dependent substrates has been reported recently [15]. This decline was attributed to a decrease in complex I activity and interpreted by the authors as resulting in dissociation of FMN from the enzyme. We were unable to see any decrease of ROS generating activity of heart SMP for at least 8 min (Fig. 5C). The simple FMN dissociation mechanism for the time-dependent inactivation of complex I during hydrogen peroxide generation proposed by Stepanova et al. [15] seems unlikely considering the high affinity of the enzyme active site for the nucleotide (oxidized and reduced) and limitation by small internal volume of mitochondria. An intriguing possibility that can be proposed to explain the discrepancy between the data reported in Ref. [15] and here is that brain mitochondria, in contrast to heart mitochondria, contain some factor(s) that binds reduced FMN with substantially higher affinity than complex I does.

When “instantaneous” rates were calculated from the progress product accumulation curves (Fig. 6B) for intact mitochondria oxidizing succinate, strong deviation from linear dependence on oxygen was seen in its low concentration area. This deviation was also evident if Eq. (4) was applied for intact mitochondria (in contrast to SMP). The reason for deviation from the first-order dependence is evidently due to inadequate response of the AR assay for hydrogen peroxide quantitation as applied to intact mitochondria, which contain NADH and other possible reductants of Res. The contribution of Res reductase activity became particularly significant at low oxygen concentration when ROS production is low. Although AR seems most widely used for quantitation of hydrogen peroxide [24], [25], [26], [27], the redox properties of Res, the key indicative component of this assay, is poorly studied. Searching the literature, we found only one short report where the redox titration of ResH_2_/Res pair midpoint potential (–120 mV at pH 7.5 versus the hydrogen electrode) was reported [50]. Resorufin reductase activity of DT-diaphorase [51], glucose oxidase [52], and microsomal NADPH: cytochrome reductase [53], [54] has been demonstrated. Respiratory complex I (Fig. 5A, curves 3 and 4, and Fig. 6A, curves 2 and 4) and likely other yet unidentified mitochondrial enzymes may also serve as the electron donors for this acceptor. The redox couple ResH_2_ (colorless)/Res (red) is reversible, its reduced form being oxidized by oxygen via a mechanism similar to that for FMNH_2_ [55], [56], [57], as evident by its SOD sensitivity (Fig. 7) and its rapid oxidation by peroxidase if hydrogen peroxide is present (Fig. 7).

The conclusions we make are following. (i) Oxygen dependence of ROS (superoxide and hydrogen peroxide) production by the combined components of heart mitochondrial respiratory chain strictly obeys simple first order kinetics within the range of oxygen concentration (0–750 μM). (ii) Concave oxygen concentration dependence of ROS production at low oxygen concentration as detected by the AR assay applied for intact mitochondria results from quantitatively inadequate response of the detecting system. (iii) Respiratory complex I and likely other intramitochondrial NADH-dependent redox enzymes are capable of Res reduction; ResH_2_ is rapidly oxidized by peroxidase. (iv) Great precautions should be taken if AR is applied to characterize mitochondrial ROS-producing activity. This also applies for other enzyme system containing low midpoint redox potential donors capable of Res reduction. The initial rates, not the amount of Res formed at some given time interval, is the reliable parameter, particularly in lower than “normal”, air saturated solution oxygen content.

## Disclosures

No conflicts of interest, financial or otherwise, are declared by the authors.

## Author contributions

V.G.G. and A.D.V. conceived and supervised the study; A.V.K. and V.G.G. performed experiments; A.D.V. and V.G.G. wrote this report.

## Sources of funding

This study was partially supported by grant 17-04-00706 to A.D.V. of the Russian Foundation for Fundamental Research. We are grateful to Administration of School of Biology for covering open access publication fee.
